# Supplementary material for: Increasing consensus of context-specific metabolic models by integrating data-inferred cell functions
Source: PLoS Comput Biol. 2019 Apr 15;15(4):e1006867. doi: 10.1371/journal.pcbi.1006867 (PMC6483243; doi:10.1371/journal.pcbi.1006867)
Supplement: S1 Text — (DOCX) [file pcbi.1006867.s001.docx]

S1 Text

**Increasing consensus of context-specific metabolic models by integrating data-inferred cell functions**

*Anne Richelle, Austin W.T. Chiang, Chih-Chung Kuo, Nathan E. Lewis*


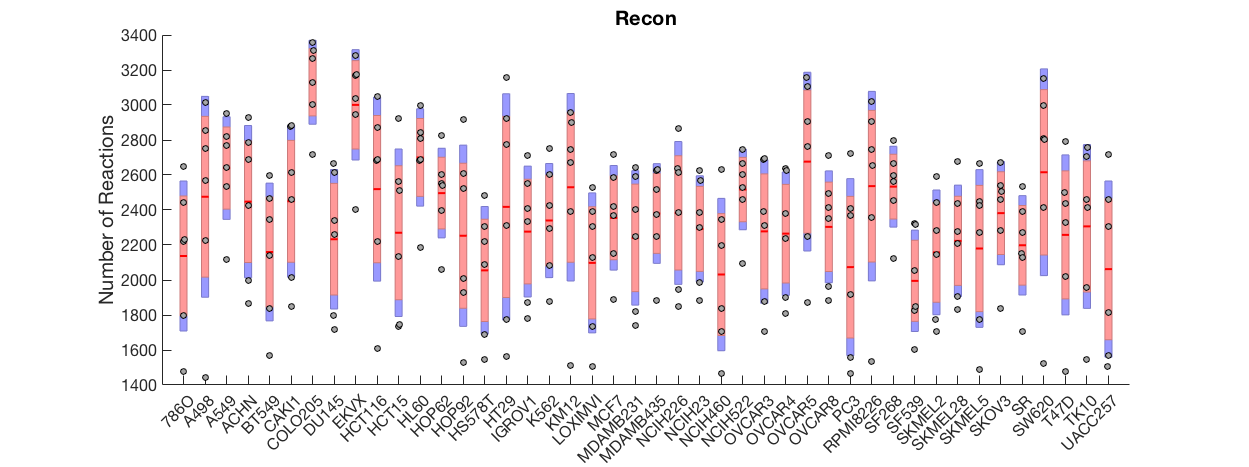


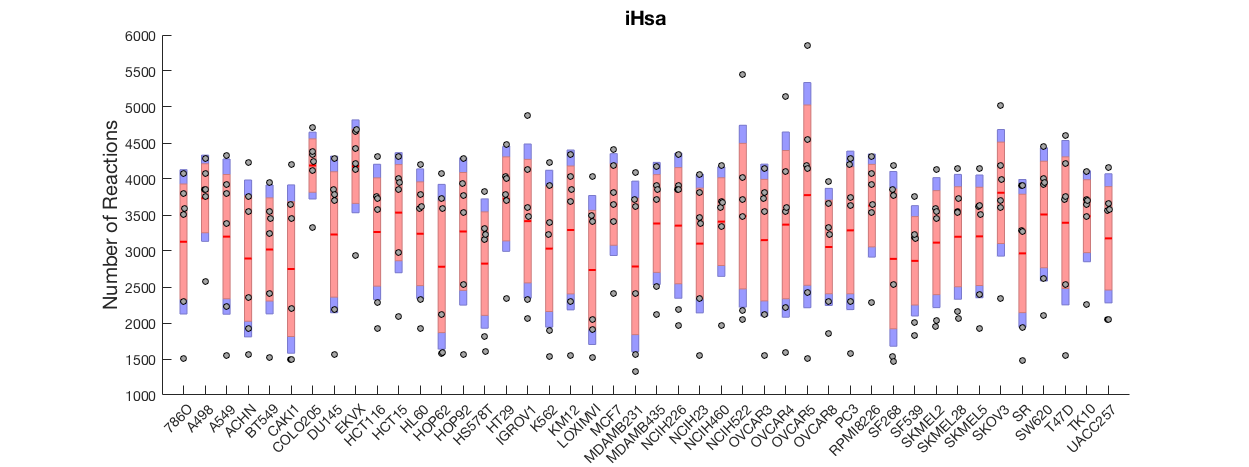


**Fig A** - The extracted cell line-specific models vary considerably in size regardless of the reference model used.


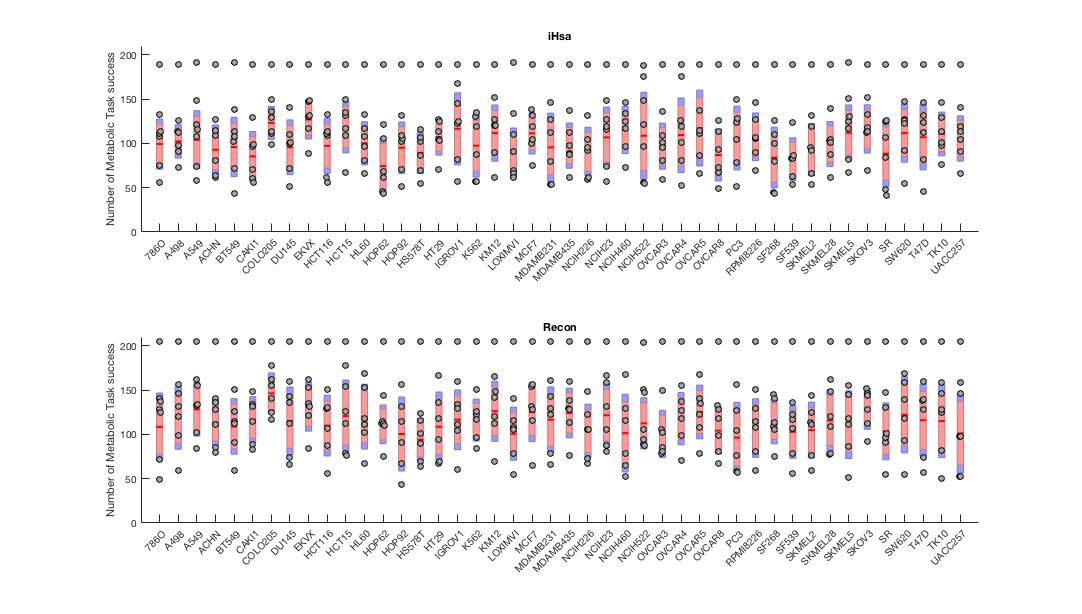


**Fig B** - The extracted cell line-specific models vary considerably in functional capacity regardless of the reference model used.

**
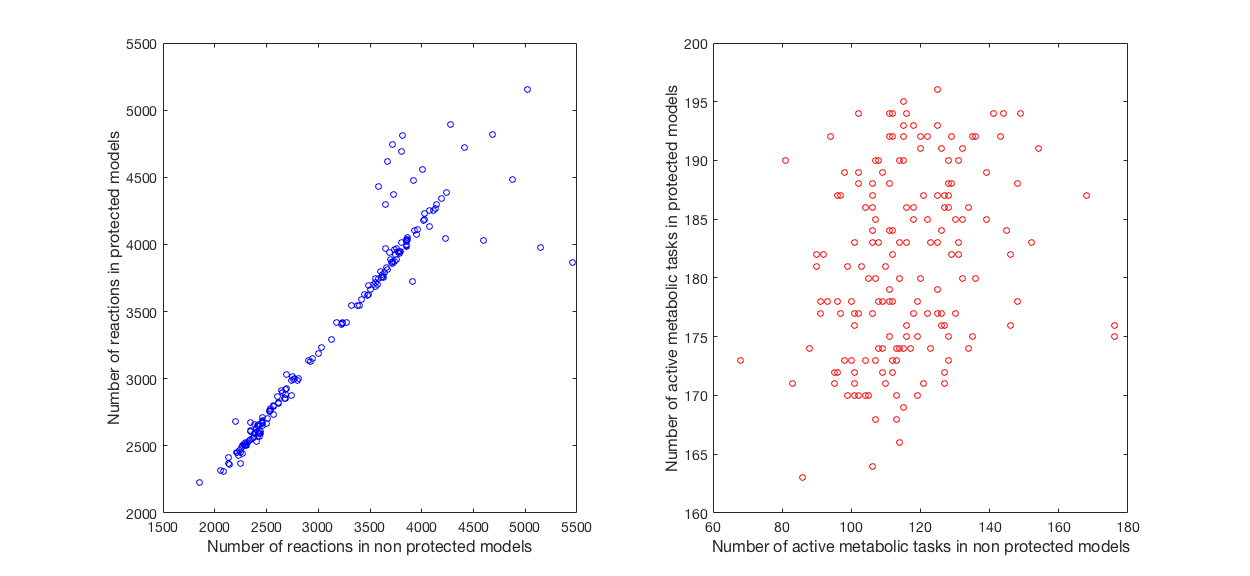
**

**Fig C** - While the number of reactions included in the extracted model does not seem to be influenced by the protectionist approach (i.e., extracted models present almost the same number of reactions for equivalent extraction setup regardless of the use of the protectionist approach), the number of active tasks is clearly higher in the models extracted using the protectionist approach.

*
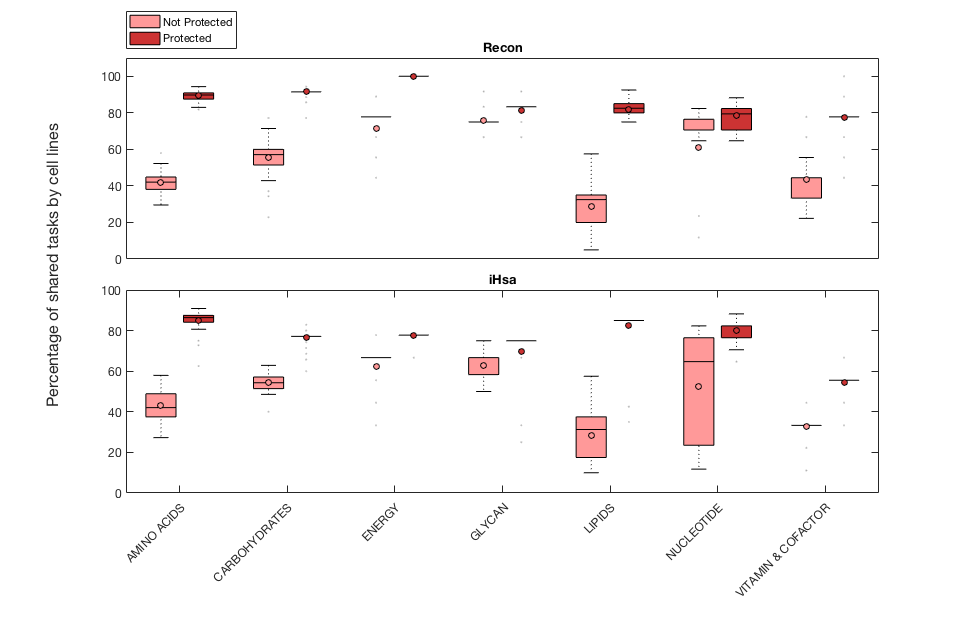
*

**Fig D** - The protectionist approach reduces the influence of the extraction method thanks to an increased number of shared tasks by cell lines that mostly benefited to the amino acid and lipid systems. Note this figure present the percentage of shared task by cell lines clustered by metabolic systems based on the models generated using only the algorithms allowing a significant protection of the inferred metabolic task (fastCORE, mCADRE and MBA).

*
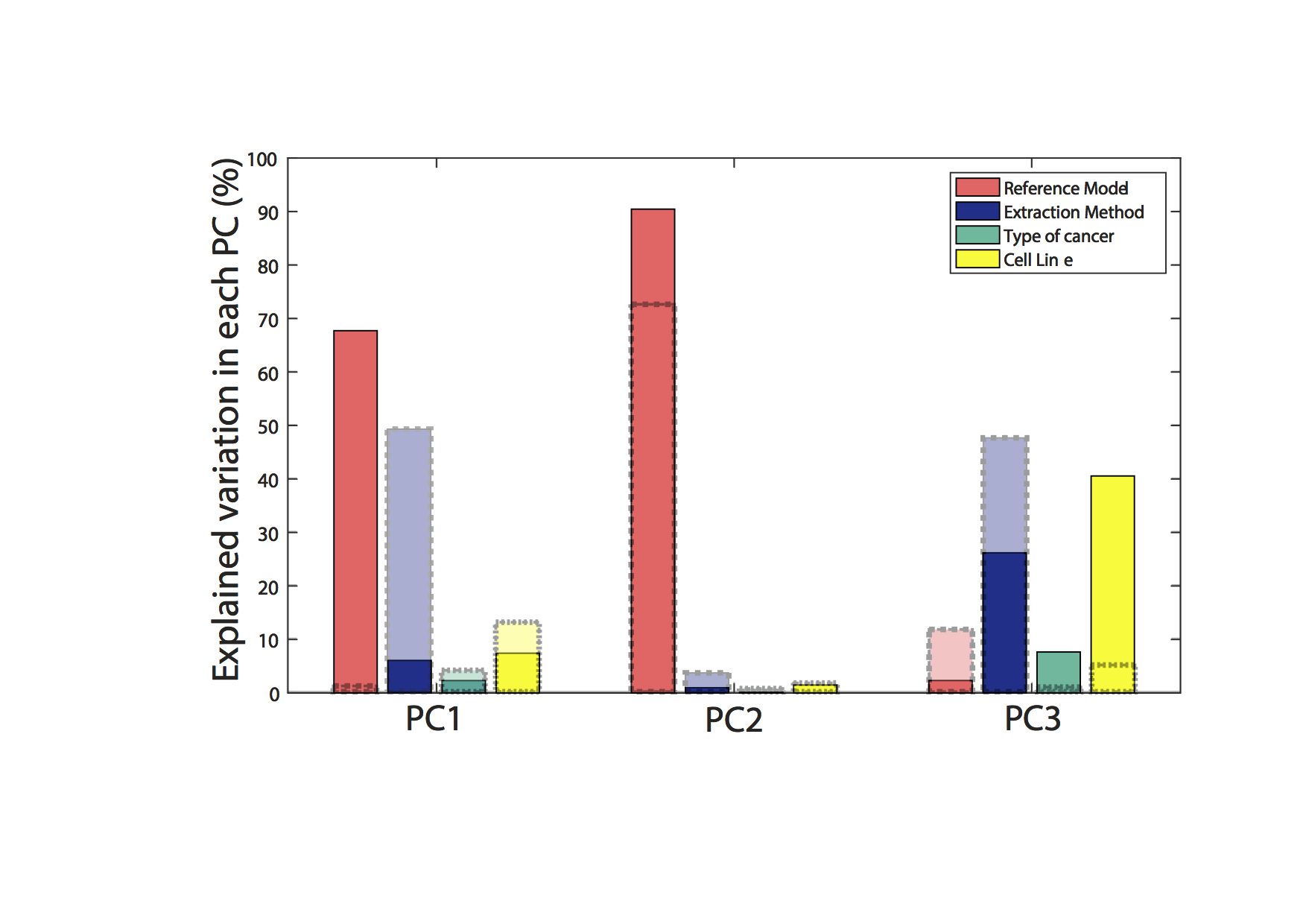
*

**Fig E** - The protectionist approach reduces the influence of the extraction method on the model functionalities but increases the relative influence of the reference model. Note this figure presents PCA analysis based on the models generated using only the algorithms allowing a significant protection of the inferred metabolic task (fastCORE, mCADRE and MBA).

**
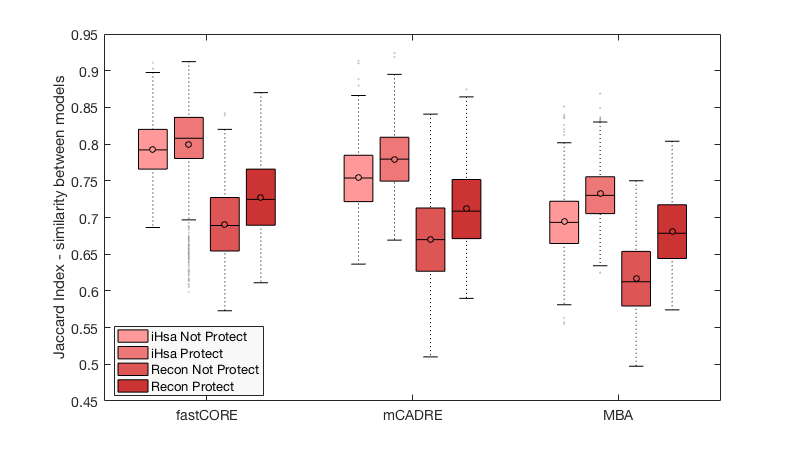
**

**Fig F** - For each method, the similarity between models of different cell lines (computed using a Jaccard index based on the reactions content) is significantly improved by the use of the protectionist approach.

**
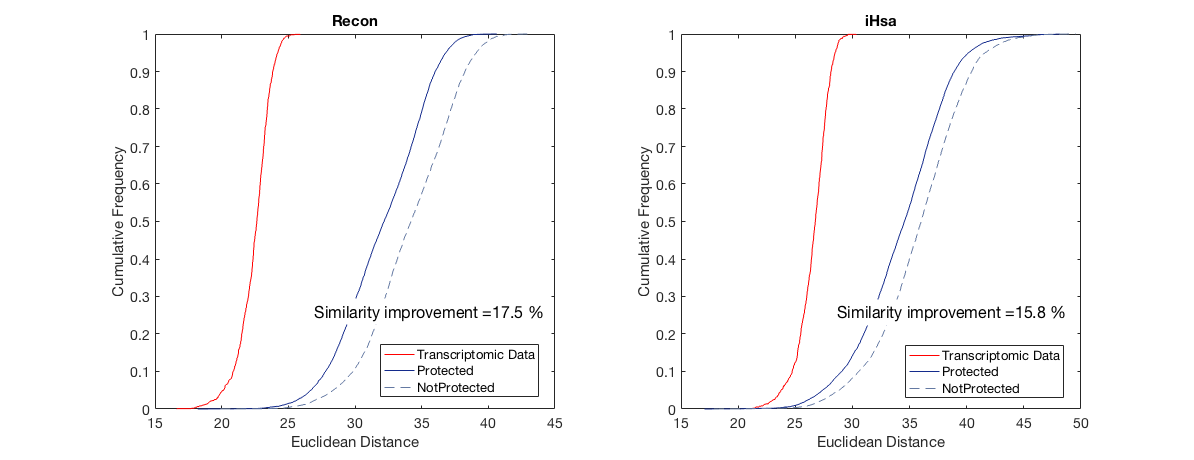
**

**Fig G** - The protectionist approach increases the similarity of models with respect to the transcriptomic data. This figure presents the cumulative frequency of measured Euclidean distances between cell-lines for 1000 randomized combinations of models selected from the 3 extraction methods (i.e. fastCORE, mCADRE and MBA) that were protected or not and compared to the Euclidean distance between cell-line observed in transcriptomic data. The similarity improvement is computed as the difference between the median distances of non-protected model and protected model with the median of transcriptomic data divided by the median distance between non-protected models and the transcriptomic data.

*
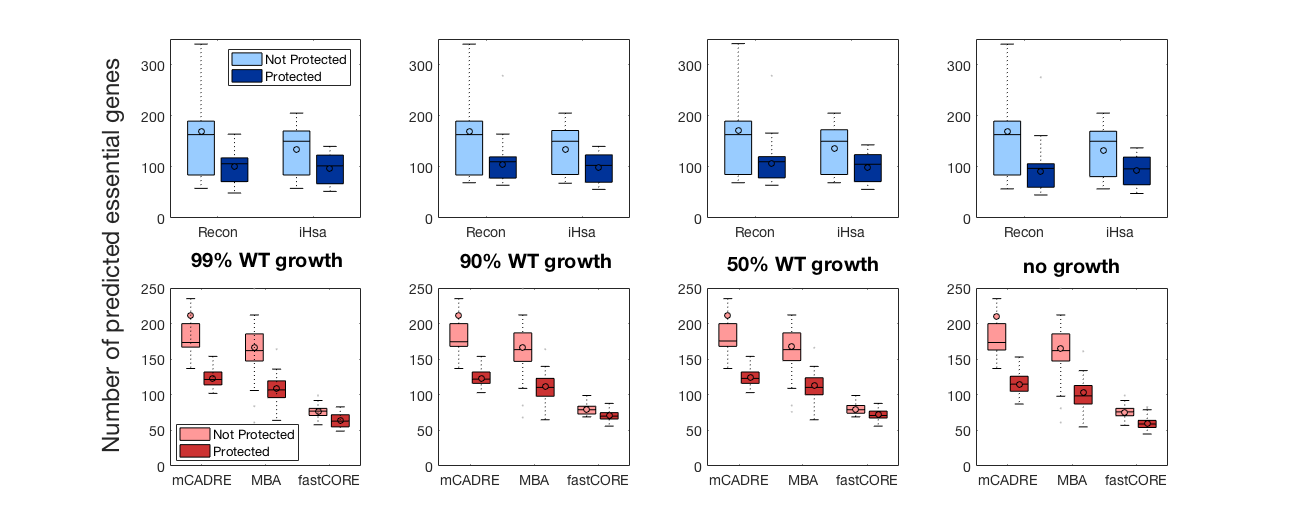
*

**Fig H** - Influence of the protection of data-inferred metabolic tasks on the number of predicted essential genes with respect to the reference model used (top) or the extraction method used (bottom) for difference choices of essentiality thresholds.

*
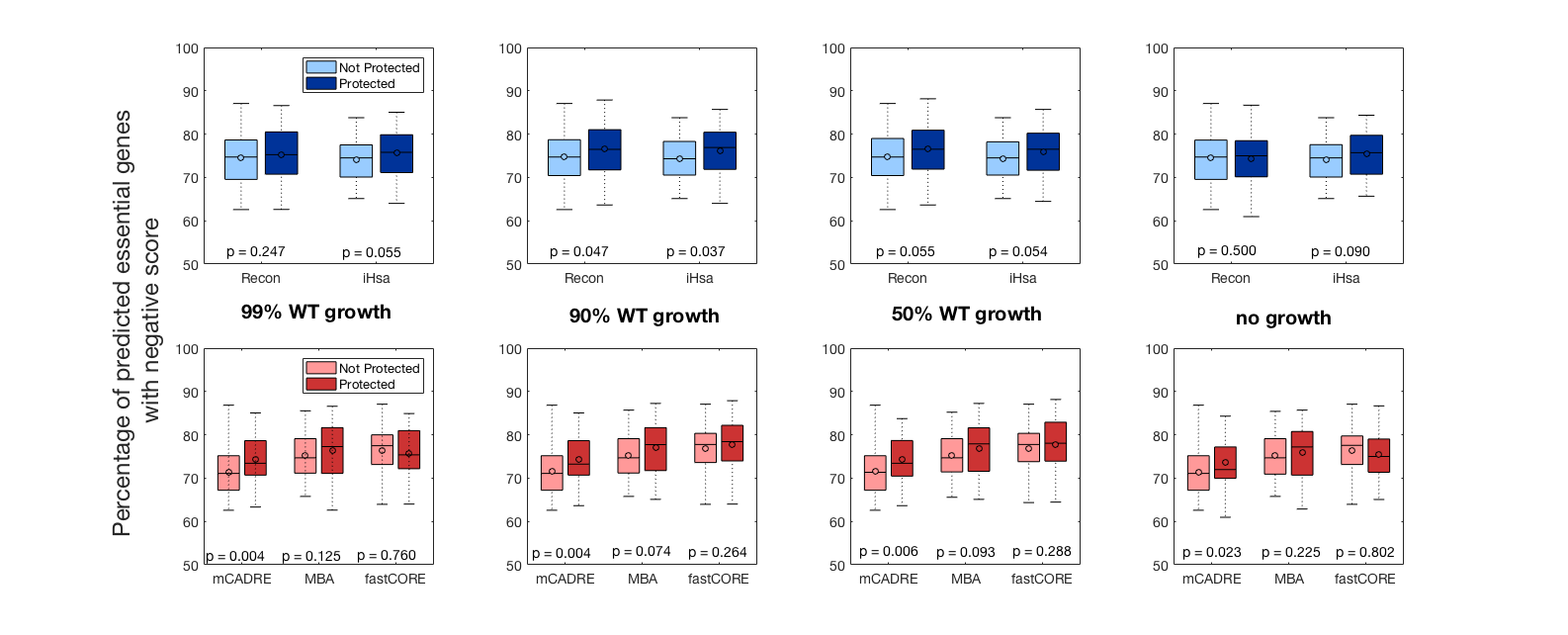
*

**Fig I** - Influence of the protection of data-inferred metabolic tasks on the percentage of predicted essential genes associated with a negative score with respect to the reference model used (top) or the extraction method used (bottom) for difference choices of essentiality thresholds and the associated one-tailed Wilcoxon p values.

*
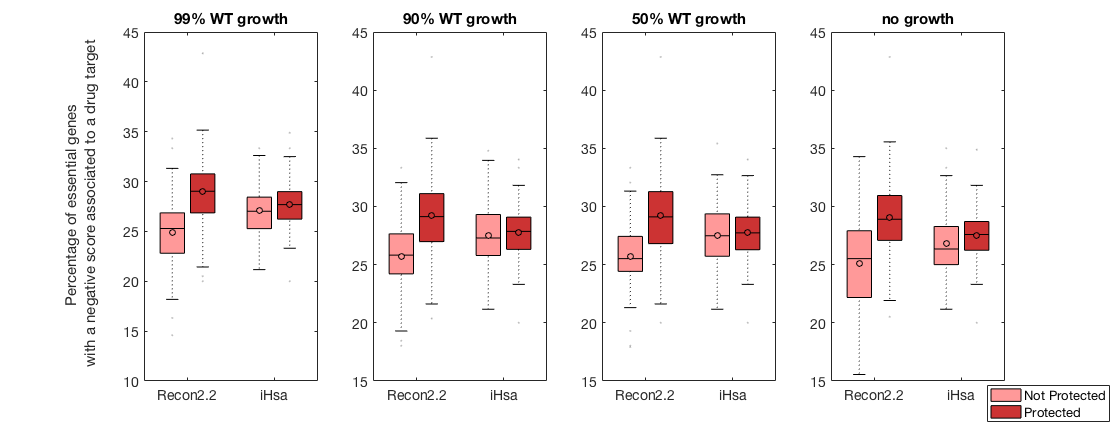
*

**Fig J** - Influence of the protection of data-inferred metabolic tasks on the percentage of predicted essential genes associated with a negative score that are associated with a known cancer drug target (S11 Table)

*
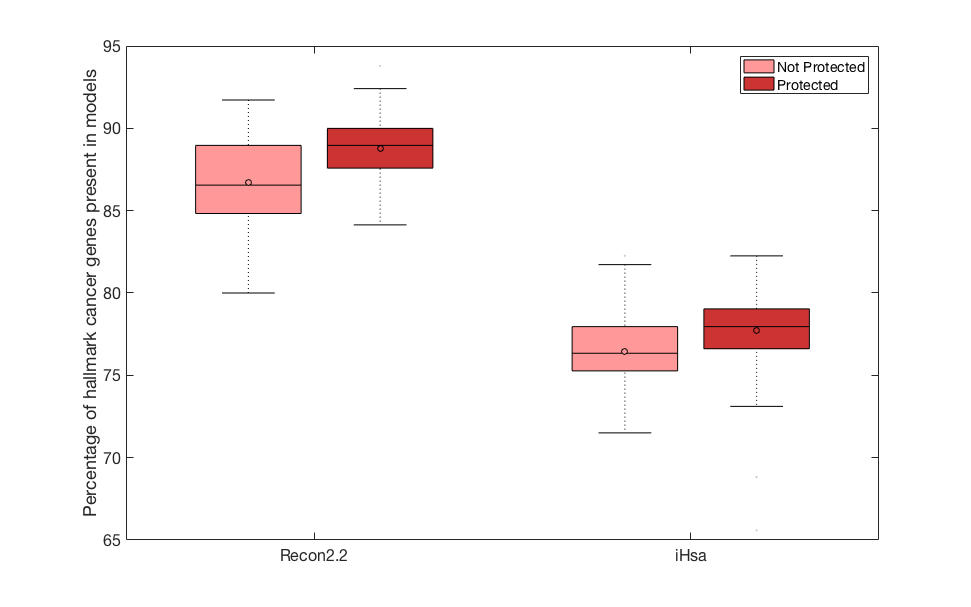
*

**Fig K** - Influence of the protection of data-inferred metabolic tasks on the percentage of hallmark cancer genes present in the extracted models (S12 Table)

**1**

**
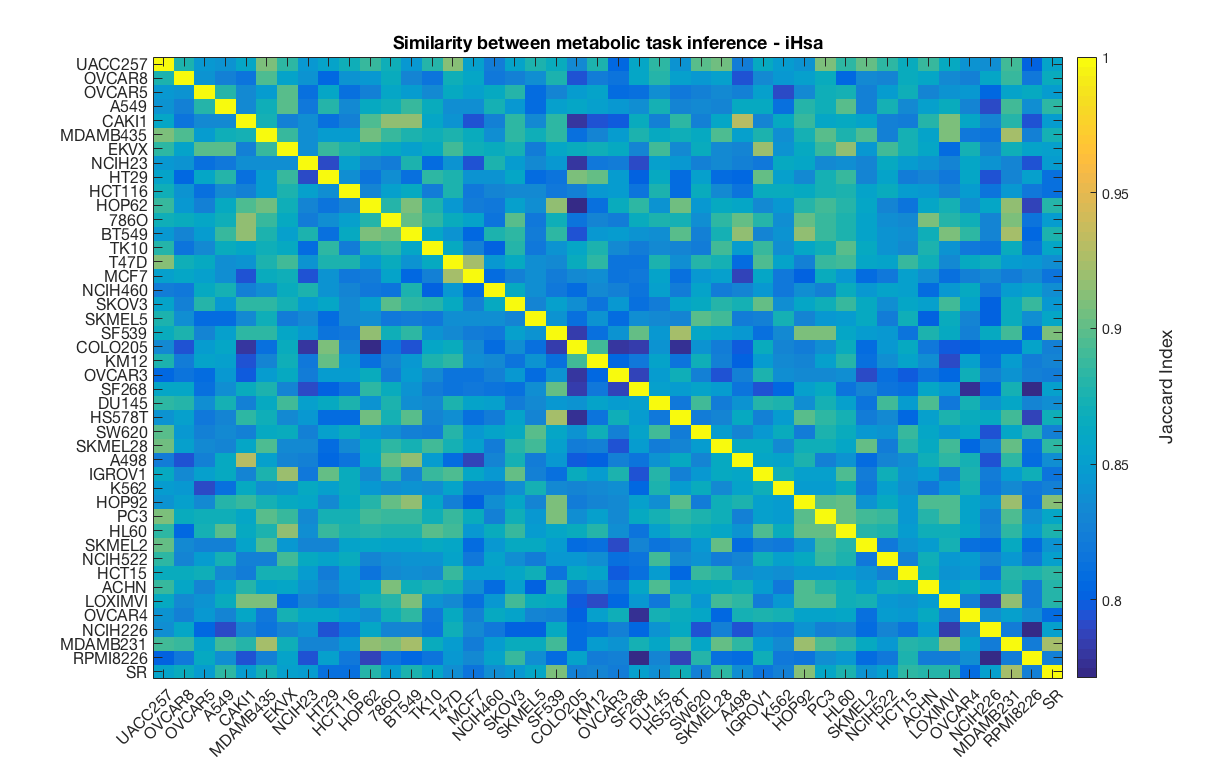
**

**2**

**
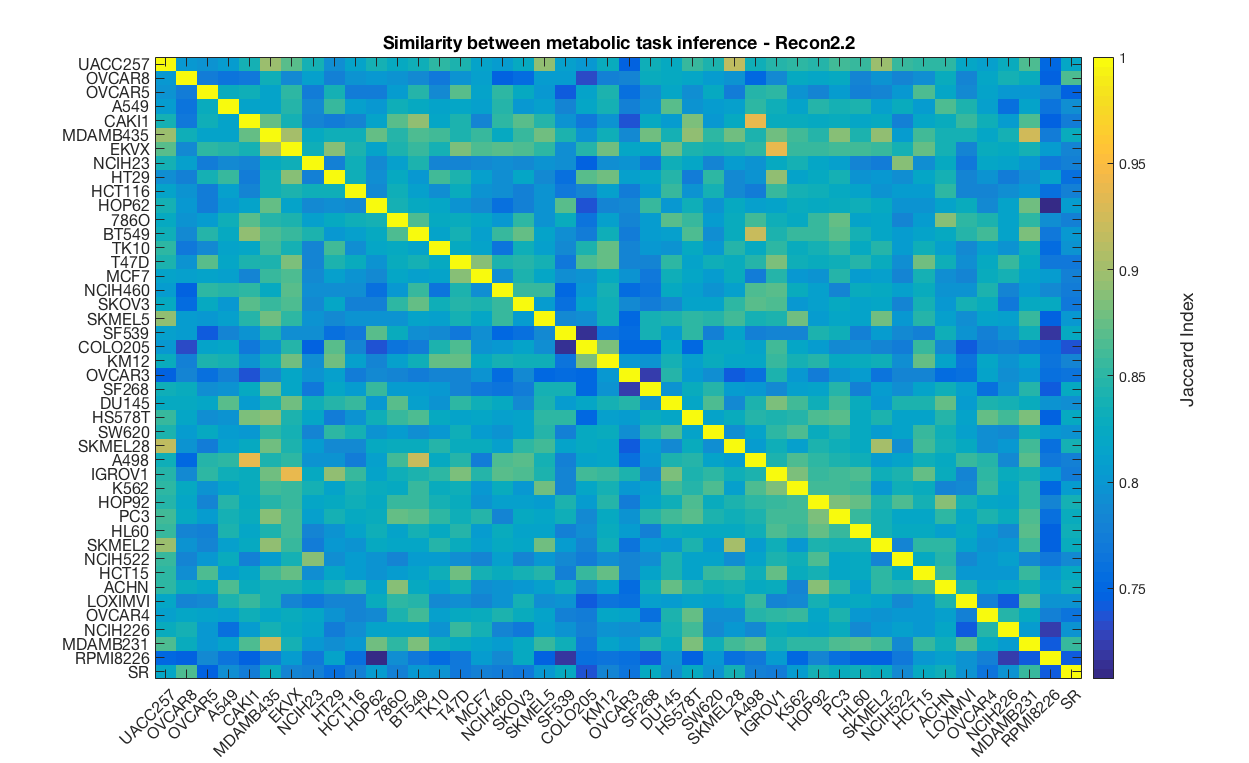
**

**3**

**
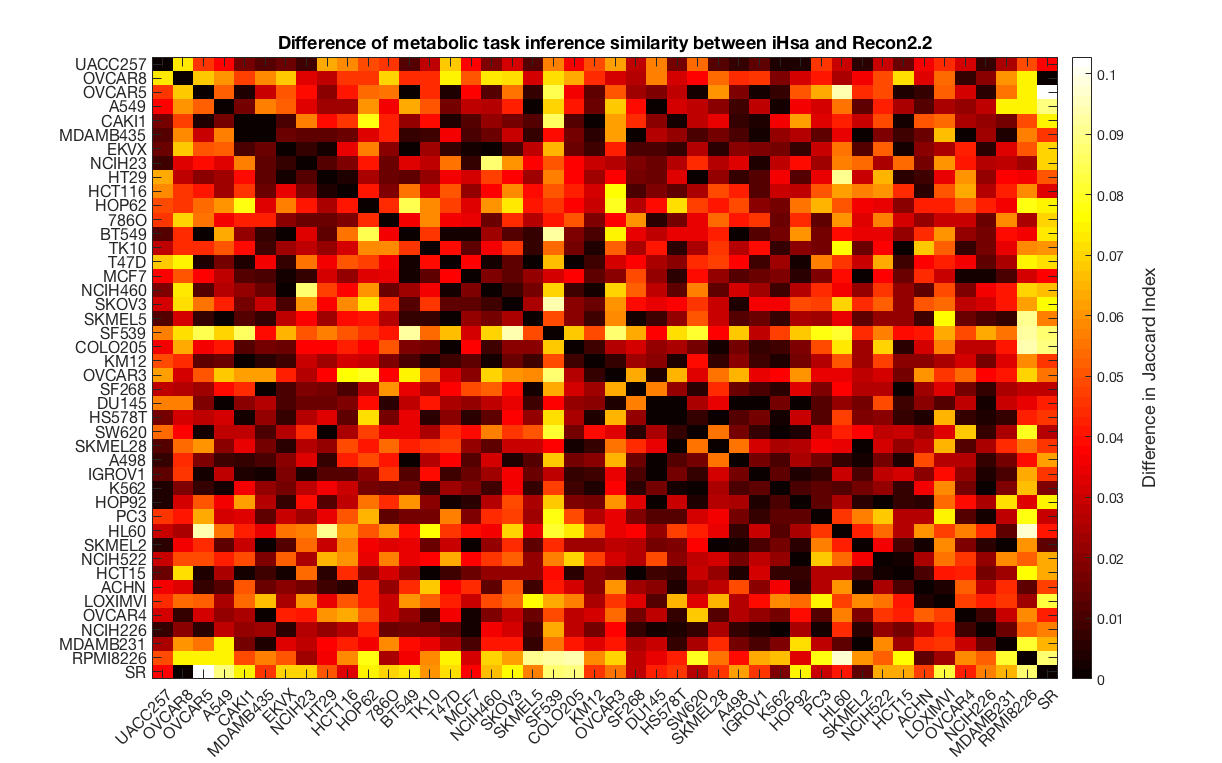
**

**Fig L -** Similarity of data-inferred metabolic tasks across cell lines. Similarity (Jaccard index) of metabolic tasks inferred using iHsa (1) and Recon2.2 (2) as reference GeM. (3) Difference between the similarity across cell lines computed for iHsa and Recon2.2
